# Butterfly Effect in Chaotic Image Segmentation

**DOI:** 10.3390/e22091028

**Published:** 2020-09-15

**Authors:** Radu Mărginean, Anca Andreica, Laura Dioşan, Zoltán Bálint

**Affiliations:** 1 IMOGEN Research Institute, County Clinical Emergency Hospital, 400006 Cluj-Napoca, Romania; anca@cs.ubbcluj.ro (A.A.); lauras@cs.ubbcluj.ro (L.D.); zoltan.balint@phys.ubbcluj.ro (Z.B.); 2 Faculty of Mathematics and Computer Science, Babeş–Bolyai University, 400084 Cluj-Napoca, Romania; 3 Faculty of Physics, Babeş–Bolyai University, 400084 Cluj-Napoca, Romania

**Keywords:** complex networks, cellular automata, image segmentation, butterfly effect, emergent phenomena

## Abstract

The exploitation of the important features exhibited by the complex systems found in the surrounding natural and artificial space will improve computational model performance. Therefore, the purpose of the current paper is to use cellular automata as a tool simulating complexity, able to bring forth an interesting global behaviour based only on simple, local interactions. We show that, in the context of image segmentation, a butterfly effect arises when we perturb the neighbourhood system of a cellular automaton. Specifically, we enhance a classical GrowCut cellular automaton with chaotic features, which are also able to improve its performance (e.g., a Dice coefficient of 71% in case of 2D images). This enhanced GrowCut flavor (referred to as Band-Based GrowCut) uses an extended, stochastic neighbourhood, in which randomly-selected remote neighbours reinforce the standard local ones. We demonstrate the presence of the butterfly effect and an increase in segmentation performance by numerical experiments performed on synthetic and natural images. Thus, our results suggest that, by having small changes in the initial conditions of the performed task, we can induce major changes in the final outcome of the segmentation.

## 1. Introduction

The various complex systems we directly or indirectly interact with, are either natural (e.g., societies, ecologies, living organisms, organs) or artificial (e.g., artificial intelligence systems, artificial neural networks, evolutionary programs, parallel and distributed computing systems). Both categories are characterized by apparently complex phenomena that emerge as a result of often nonlinear spatio-temporal interactions among a large number of elements at different levels of organization [1]. Such a complex system is a collection of simple units (elements, entities, agents) that interact, leading to emergent outcomes that are often difficult to predict simply by investigating the individual interactions. Cellular automata (CA) [2] represent important tools in the study of complex systems and they are being successfully applied in a variety of domains, including image segmentation [3].

However, we believe that image segmentation does not yet benefit from all advantages of self-organization and emergence potentially occurring in cellular automata, as in current approaches, these computational methods are mostly used as a parallelization tool. This motivates the study of image segmentation methods that build upon cellular automata on the edge of chaos, where remarkable phenomena occur in complex systems.

In this paper we aim to optimize the GrowCut image segmentation algorithm [3] by introducing a new neighbourhood system. We analyze the GrowCut algorithm at the cellular automata level and propose an improvement to the algorithm based on prior work in cellular automata [4,5,6,7]. This can uncover a butterfly effect: a small change in the automaton specification can determine qualitative changes in the algorithm output. Moreover, the neighbourhood topology can influence the speed of the searching process, positively impacting segmentation performance. The investigation of the classical topologies have revealed that the simple von Neumann neighbourhood can not escape from local optima, while Moore neighbourhood could overpass this problem.

The size and the topology of the neighbourhood, and implicit of the CA’s grid, have a great influence on the behavior of the system. Different neighbourhood topologies (lattice, network, small-worlds) have been investigated in order to study their influence on the rule performance. In [8,9,10,11,12,13], the neighbourhood topologies are evolved for cellular automata. In these studies a better performance of evolved topologies over the regular lattices in CA majority and synchronization tasks was shown.

Furthermore, enlarging the neighbourhood could weakly improve the segmentation result, but the added computational effort does not always justify this performance gain [4,5]. Based on this previous analysis, we intend to enrich the classic neighbourhoods by new elements: some remote neighbours which are situated at a given distance to the main cell. These outside neighbours could be disposed in various places: pure randomly (obtaining a star-based topology—like in [4,5]) or controlled randomly (inside a layer or a band of a given width circumscribed to the classic neighbourhood—as we investigate in this paper). The resulted neighbourhood’s topologies accelerate the information propagation and the region growing involved in the segmentation flow.

Moreover, the neighbourhood extension affects the emergence of the underlying CA, enriching the semantic of the global behaviour of the GrowCut algorithm: a small perturbation (in terms of considered neighbours) can stimulate in a non-linear manner the segmentation according to the complex system, giving rise to a butterfly effect. In this case, small events can be viewed as catalysts changing the initial state of the system. Finally, these small changes also positively affect the performance of the cellular automaton-based system.

In order to provide an overview of the field, in the beginning of the paper, the reader is familiarized with the domain of complex systems in Section 2.1 and cellular automata in Section 2.2. An introduction to the problem considered for validating our approach and its relation to cellular automata is given in Section 2.3. Our proposed cellular automaton for image segmentation is afterwards described in Section 3, with supporting numerical experiments in Section 4 and concluding remarks in Section 5.

## 2. Theoretical Background

Cellular automata are ideal tools for modelling both natural processes (e.g., physics, geology, chemistry, biology, etc.) and complex systems (studying abstract notions of self-organization and emergent computation in complex systems).

### 2.1. Complex Systems, Emergence and Self-Organization

A complex system has a large number of elements that are arranged in structures, forming topologies which can scale very well. These elements interact locally–each one is connected, directly or indirectly, with other elements. This enables the elements and the associated structures to change, evolve, or adapt to particular environments or problems, giving rise to complex system dynamics [14]. These dynamics are influenced by many factors, like number of elements and their interaction patterns, the organizational structure of the system (e.g., groups, hierarchies), or feedback loops. Complex systems have three important properties:

- They are robust to outside input and are able to self-organize [1].
- They can behave chaotically: the (highly) non-linear dynamics of some systems can make them very sensitive to initial conditions, making them unpredictable.
- They show emergent properties. Here, “emergence” refers to the fact that the whole is not the sum of its parts [15] and occurs when the behavior of a system (at the macro– or global level) cannot be reduced to the sum of the behavior of its parts (at the micro level) [15,16].

We have a special interest about the last two properties. An interesting phenomenon, called butterfly effect, can be observed in non-linear systems: a small change in initial conditions can result in vastly disproportionate response at a later stage [17]. In this context, it is difficult to model the chaotic behavior numerically. Related to the emergence process, we are interested in the micro-macro effect [16,18] that refers to those characteristics which become visible at a higher, macro level and that cannot be explicitly identified at the lower, micro level.

### 2.2. Cellular Automata-Prerequisites

Cellular Automata (CA) represent useful and important tools in the study of complex systems and interactions. Originated from John von Neumann and E. F. Codd in 1950s [19,20] and popularized by Conway’s Game of Life in 1970s, CA can model complex computations. The formalism behind CA refers to spread collections of simple and locally interacting elements which evolve following a set of rules, emerging into a global behavior.

**Figure 1:**
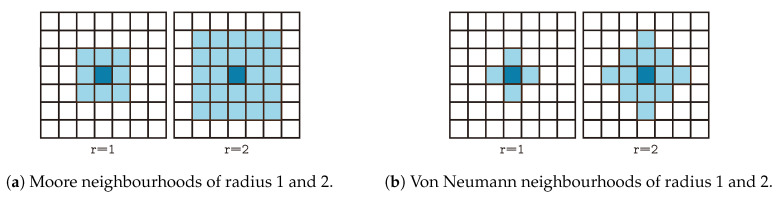
Examples of Moore (**a**) and von Neumann (**b**) neighbourhoods.

A CA is represented by a set of cells disposed on a grid, and a set of transition rules. At each point in time, cells are in a given state, evolving from an initial state. The rules dictate how cells change their state locally, based solely on the current state and the states of the neighbouring cells. The rules are applied simultaneously for all cells, at each time step. A CA neighbourhood system determines the connectivity pattern between each cells and the other cells in the system [21]. The two most popular such neighbourhoods are the Moore and von Neumann neighbourhoods [19,22], which are exemplified for 2D grids in Figure 1.

More formally, a CA is a triplet (S,N,δ), where *S* is a set of possible states, *N* is a neighbourhood system, and $\delta :S^{N}\mapsto S$ is a transition function, which formalizes the transition rules. The 2D von Neumann neighbourhood of radius *r* over a grid *Z* is formalized as:

(1) $$N_{V}^{r}\left(x_{0},y_{0}\right)=\{\left(x,y\right)\in Z:|x-x_{0}|+|y-y_{0}\left|\leq r\right\}$$

Similarly, the 2D Moore neighbourhood of radius *r* over a grid *Z* is formalized as:

(2) $$N_{M}^{r}\left(x_{0},y_{0}\right)=\{\left(x,y\right)\in Z:|x-x_{0}|\leq r,|y-y_{0}\left|\leq r\right\}$$

CA, as computational models, are discrete in both space and time (each cell evolves from a state to another), homogeneous in space and time (all changes of the cell’s state always follow the same rules) and local in their interactions. Taxonomically, there are various classifications of CA, based on grid dimensionality (e.g., 1D, 2D, *n*D), state space (e.g., binary, multi-state CA), or size and topology of neighbourhood system. It is noteworthy that some particular local phenomena appearing in CA depend on the size and the topology of the neighbourhood [23].

From a system’s behavior point of view, Wolfram [24] has investigated the rules of 1D and 2D binary CA and he has identified four classes of CA:

- Class 1: CA which evolve to a single, unique homogeneous state for all cells, irrespective of the initial state;
- Class 2: CA displaying simple separated periodic structures that endlessly cycle through a fixed number of states;
- Class 3: CA displaying chaotic or pseudo-random aperiodic structures;
- Class 4: CA which yield complex patterns of localized structures and are suitable for universal computation.

Cellular automata that exhibit some of the most interesting behaviors are those belonging to classes 3 and 4. In class 3 CA any change in the initial conditions is communicated globally and visible in the later iterations of the evolution-the so-called butterfly effect. In class 4 this kind of propagation is also possible but often affects only parts of the system. However, especially when talking about chaos and complexity, despite many attempts reported in the literature of finding a way to decide the membership of a CA to a certain class, this issue remains undecidable [25].

### 2.3. Cellular Automata and Image Segmentation

Image segmentation refers to the task of partitioning an image in sets of regions such that the regions have similar meaningful characteristics (e.g., they represent the same object of interest). Each pixel of the image is labelled so that pixels with the same label share some common features (e.g., they belong to the same organ/bone/blood vessel in a medical image).

The segmentation of an image, *I*, can be defined as the partitioning of *I* in *L* sub-regions, such that:

- The union of all regions sums up the original image;
- Each pixel of the image is assigned exactly one of the *L* labels (each label corresponds to a region).

Image segmentation can be perceived as a clustering problem: it takes unlabeled data (i.e., pixels or voxels) and divides it into two or more clusters representing objects and background.

Cellular automata can be successfully applied to the task of image segmentation [3,26]. The cellular automata used for image segmentation are usually 2D, where each pixel maps to a cell of the CA. The state of a cell contains information about the labelling of the corresponding pixel, and changes at each time step based on the state of the neighbouring cells.

Advantages of using CA for image segmentation:

- It is an unsupervised method: it does not require a training set containing ground truth information.
- It is highly parallel: computing the next state of a cell depends only on the current state and neighbouring cells’ state.
- For every new image a CA is constructed and evolved in order to identify the corresponding segments, i.e., no prior training is required. A large number of iterations required to obtained a segmentation can be view as a drawback, but the possibility of implementing a parallel version of the CA mechanism counteracts it.

One of the most popular approaches in the literature for CA-based image segmentation is given by the GrowCut algorithm introduced in [3]. The algorithm has many advantages: it can segment images of any dimension, it can solve hard segmentation tasks, it is extensible, it works with multiple labels, it is simple, and it is interactive. This is a seed-based approach, i.e., it lets the user select a set of pixels belonging to the desired regions (e.g., background and object).

The algorithm defines the state of the underlying CA’s cells to be a triplet (λ,σ,C) where λ is the label of the cell, σ is its strength and *C* is a feature vector (e.g., grey intensity). At each time step, labels (initially described just by the seeds) are propagated locally: each cell updates its label and strength of the label according to a “similarity” function between the cell and its neighbours. This labeling process continues until the automaton converges to a stable configuration.

While GrowCut has been extended in various directions, its classical version is still being successfully applied to image segmentation tasks, especially in the medical field, either as the main segmentation method [27,28] or as part of a larger pipeline [26,29,30]. This is the case since, as a segmentation algorithm, GrowCut provides several desireable qualities such as interactivity, small fine-tuning overhead, and relatively clear segmentation semantics. To the extent of our knowledge, extensions to the algorithm have either traded-off some of these qualities for better performance, or specialized the algorithm to a niche task.

In [31], the authors present an extension of the automaton’s update rule by computing cell’s strength by means of a fuzzy gaussian membership function. As a consequence, the algorithm becomes more robust towards the seed selection and it even eliminates the need for specifying background seeds, while also being more specialized towards unclear segment delimitations. The method’s good performance was validated on mammography images, where handling of unclear boundaries is of paramount importance.

In [32], the authors propose a means of automating the GrowCut algorithm by designing simple, high-recall models of the object of interest. These types of models can then be used to generate a small number of seed pixels which are fed into the GrowCut algorithm. In this case, the proposed extension gives up a desireable quality of the algorithm (interactivity and fine-tuning overhead), while maintaining the algorithm’s generality.

In [6,7,33], a series of fully-automatic extensions of the GrowCut algorithms have been proposed. While these show general and good performance relative to automatic and unsupervised algorithms, they give up all of the classical GrowCut’s qualities: interactivity, ease of setup, clear semantics.

Due to the dimension-agnosticism of the underlying CA, GrowCut can be extended to multidimensional objects. In [34], the author presents a mathematical framework for defining standard neighbourhoods with arbitrary density, that has as extreme cases the multidimensional Von Neumann (sparse) and Moore (dense) neighbourhoods. Moreover, the paper formalizes neighbourhoods of arbitrary radii, which are relevant in our approach to include long-distance information in CA-based image segmentation applications.

Finally, previous studies on GrowCut algorithm and classical neighbourhoods [4,5] have shown the impact of the neighbourhood topology on the final outcome. All these results are indicators of how novel topologies and neighbourhoods can trigger good performance in CA tasks and can be adapted to improve segmentation techniques.

## 3. Proposed Cellular Automata for Image Segmentation

The purpose of our contribution is to bring cellular automata closer to the edge between order and chaos, where notable phenomena occur, with direct application to image segmentation. We start from the popular GrowCut algorithm (see Section 3.1 for a description of the algorithm and the notation used). Analyzing this algorithm at the CA level enables us to extend it using insights from the field of cellular automata.

Specifically, we extend GrowCut with neighbourhoods which contain, alongside the regular neighbours, several remote cells that are disconnected from the main neighbourhood. These distant cells can bring additional information about the image segments which will propagate through the entire CA evolution. This hybrid neighbourhood combines local and global information in order to help the system’s convergence.

### 3.1. GrowCut

We further detail the original GrowCut algorithm, first introduced in [3]. This region growing method starts with a grid of cells, where some have been labeled in advance (by a user or in an automatically manner). The states of the CA cells are described using a triplet (*label*, *strength*, *feature*). The labels corresponds to the regions to be segmented, the strengths are controlled by the algorithm (the initial labelled cells have strength 1, on a continuous [0–1] scale), while the features could have various semantics. In image segmentation, a possible feature is the pixel intensity, which can be an integer on a 8-bit, 12-bit or 16-bit scale. From the initial labels, the regions start to grow around them, the expansion process being guided in the direction of minimum feature difference.

The transition rule for GrowCut’s CA acts as a soft label propagator: A cell *p* will inherit a neighbour’s *q* label (or, alternatively, *q* will capture *p*) whenever *q* is stronger than *p*. This relation is formalized by Equation (3):

(3) g(||C(p)−C(q)||)·σ(q)>σ(p)

where C(·) is the feature vector associated with the cell, σ(·) is the strength of a cell, and g(·) is a non-decreasing function bounded to [0,1]. In our experiments, C(·) is the grey level of the pixel represented by the cell.

Whenever a cell *p* is captured by a neighbour *q*, the label of *q* is propagated to *p* (i.e., λ(p)←λ(q), where λ(·) is the label of a cell). Moreover, the cell *p* also changes its strength (i.e., σ(p)←g(||C(p)−C(q)||)·σ(q)). Thus, the strength parameter captures the confidence that a cell has a particular label. It is worth noting that the most popular neighbourhoods used in GrowCut are the Moore and von Neumann neighbourhoods of radius 1 [19,22].

### 3.2. Band-Based GrowCut (BBG)

The proposed algorithm extends the classic GrowCut by a new neighbourhood topology. This extension consists of a band of distant neighbours (called remote neighbours), possibly unconnected to the classical ones. We define a neighbour band spanning the radii *a* through *b* as:

(4) $$N_{BB}^{\left(a,b\right)}\left(x_{0},y_{0}\right)=\{\left(x,y\right)\in Z:a\leq |x-x_{0}|\leq b,a\;\leq |y-y_{0}\left|\leq b\right\}$$

**Figure 2:**
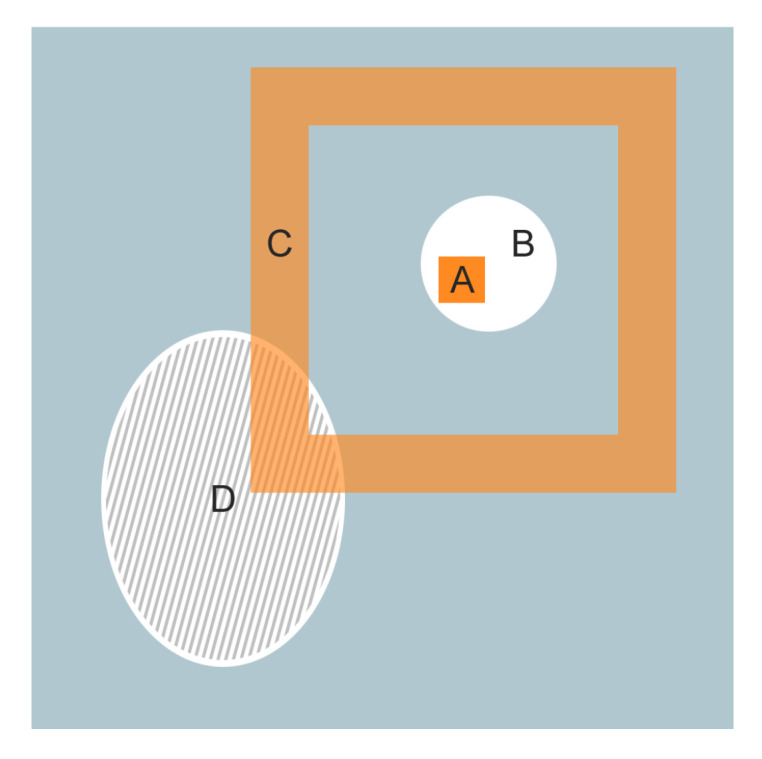
Example of a scenario where a band-based neighbourhood captures information that is not local enough for the classical neighbourhood. A: The current cell analysed by the algorithm; B: foreground object which contains no seed pixels; C: remote neighbourhood of cell A; D: foreground object which contains seed pixels.

In Figure 2, we present a situation in which the band-based neighbourhood captures information that is not local enough for the classical GrowCut neighbourhood. This example illustrates one of the limitations of GrowCut that we are addressing by using band-based neighbourhoods. Specifically, we have to identify an object that is split into multiple unconnected regions (B and D). If the seeds are supplied only for one of the main regions of the object (B or D), classical GrowCut will be unable to propagate labels between the two regions, in spite of substantial similarity.

In our example, we assume that foreground seeds are located only within the D region (Figure 2 shaded area). Because all pixels surrounding region D are background, in classical GrowCut there is no path through which the seeds can propagate their label to region B. When using remote bands (region C in Figure 2), though, cell A can receive labels from region D through a remote neighbour.

**Figure 3:**
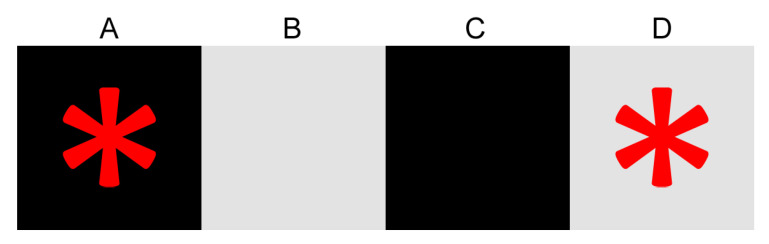
Example image used for showcasing label propagation in GrowCut. (**A**–**D**) are individual pixels. Asterisk denotes a seed pixel.

From a theoretical point of view, this discrepancy in the ability of segmenting unconnected regions occurs due to the way the labels propagate in the two GrowCut flavors. Figure 3 illustrates an example image that showcases the inability to propagate labels using standard neighbourhoods. In this example, pixels A and D are set as background and foreground seeds, respectively. The initial states of the cells in GrowCut’s CA are detailed in Table 1.

After the first iteration, A will propagate its label to B, and D will propagate its label to C. Table 2 shows the strengths of the attacks of neighbours against cells in the entire CA. The successful attacks, resulting in label propagation, are emphasized in bold. Thus, the first iteration resulted in mislabeling the B and C pixels. The state of the cells after this iteration are detailed in Table 3.

At this point, GrowCut has converged (Table 4 shows the strengths of the attacks in the second iteration). We can see that, due to the limited reachability of the standard neighbourhoods, the algorithm ended up mislabeling the B and C pixels.

From an implementation point of view, our proposed algorithm extends classical GrowCut only with respect to the considered neighbourhood. Thus, at each iteration of the algorithm for each cell, we also consider the *extended* neighbourhood (the band-based one) $N^{\left(a,b\right)}\left(\cdot \right)$. This is done after considering the original neighbourhood, such that the original neighbours win in case of a tie.

Since, for each cell, the algorithm will compare against all neighbour cells, our extension can quickly become intractable as the width and distance (to the cell) of the band grow. Specifically, the number of cells in a *d*-dimensional Moore neighbourhood band $N^{\left(a,b\right)}$ spanning the radii *a* through *b* is stated in [34] to be:

(5) $$|N^{\left(a,b\right)}{|=\left(2b+1\right)}^{d}-{\left(2a+1\right)}^{d}$$

Equation (5) illustrates the rate of growth of these band-based neighbourhoods, and suggests that considering all of these remote neighbours in each iteration of the algorithm can become intractable. To address this problem, we propose a scheme involving sampling *k* such remote neighbours at each iteration, for each cell (the sampled neighbours are different for each cell). We will denote such neighbourhood as $N_{k}^{\left(a,b\right)}$.

This scheme is motivated by the fact that remote neighbours contain highly variable information, which are not always relevant to the current cell’s labeling. If information is relevant, though, we would like it to be both spread around the neighbourhood and persistent across time. For this reason, our scheme has a vey high probability of finding this information if independent samples are drawn for each cell, at each iteration. In Section 4.3.1 we show experimentally that Band-Based GrowCut is robust against the choice for *k*. Moreover, the original hybrid neighbourhood, proposed for 1-dimensional CA [35] employed a similar random sampling of neighbours, which was validated experimentally to be robust to dynamic resampling.

### 3.3. Theoretical Analysis of BBG

Effects on convergence: Changing the neighbourhood of the CA does not affect the convergence of the algorithm. GrowCut has been shown to be equivalent to the Ford-Bellman algorithm [36]. In the equivalence proof, the number of neighbours has no effect on the development of the proof.

Higher-dimensional images: The algorithm and neighbourhood specifications are agnostic on image/grid dimensionality. In practice, however, the dimensions of the image might not have the same semantics, e.g., in medical images, multiple consecutive 2D slices are packed into a 3D volume, but a step along the third dimension discards information available between the slices. In such situations, the neighbourhoods used need to be adapted to fit the application, e.g., by rescaling along a dimension, using slice-based neighbourhoods.

Emergence: The purpose of proposed neighbourhoods is to check whether the pixels situated outside the current cell’s immediate vicinity can influence the results of the segmentation task. In this context, remote neighbours’ (desired) effect is to influence the state of the cell using global information.

From a systems point of view, GrowCut is the underlying CA that governs the dynamics of the system. GrowCut can grow spatial regions of cells with identical labels—a simple emergent process belonging to class 2 of CA, known as synchronization [37]. As the CA evolves, the majority of cells from such a synchronized region can either maintain their states or show a periodic pattern. The cells that do not subscribe to these simple dynamics are located on the edges of the synchronized regions. These cells govern the evolution of the synchronized region, by increasing/decreasing its volume. Thus, the contribution of edge–cells in this process is enhanced by including remote bands in the neighbourhood.

In the context of image segmentation, a synchronized region corresponds to a segment of the image (foreground or background). The proposed neighbourhood will influence the growth or the shrinkage of these segments (when the neighbourhoods of cells located on the segment edge become more distant, the segment will expand and viceversa). The proposed approach can create new regions because of remote neighbours (as opposed to classical GrowCut).

## 4. Numerical Experiments

In order to assess the performance of classic GrowCut and Band-Based GrowCut (BBG), we performed a set of quantitative and qualitative experiments. For all experiments, we compared different neighbourhood configurations of BBG as well as the classical GrowCut neighbourhoods.

### 4.1. Data

We ran experiments on synthetic, real-world and medical data, covering both 2D and 3D scenarios. We describe below the used datasets:

#### 4.1.1. Synthetic Images

We designed a set of 10 synthetic images featuring different types of noise (denoted as Synthetic). The purpose of this dataset is twofold: on one hand, it’s simplicity enables us to better understand the dynamics of the algorithm; on the other hand, it was used to gather numerical insights into the butterfly effect of the underlying CA.

#### 4.1.2. Real-World and Medical Data

We ran experiments on a randomly selected subset of 100 2D images from the Berkeley dataset [38] (denoted as Berkeley). The dataset contains natural images, lacking a specific theme.

For 3D images, we used the MMWHS dataset [39], a dataset of clinical MRI and CT scans of the human heart. We used 20 MRI scans of this dataset (the mr train subset), along with ground truth volumes that segment the heart from the rest of the scan (denoted as 3D MRI). We have also ran experiments on a 2D projection of the (same) MRI data, by selecting the most representative slices (denoted as 2D MRI). For all datasets, we simplified the segmentation task to foreground/background segmentation by considering all labeled objects as foreground.

### 4.2. Methodology and Setup

In this batch of experiments, we explored the performance of the different proposed neighbourhood systems, as measured not only by standard image segmentation metrics (e.g., Dice Coefficient, Hausdorff Distance), but also by other metrics related to the complexity of the proposed approach.

In all of our experiments only Moore neighbourhoods were considered. We note that the classical radius 1 Moore neighbourhood is part of every BBG configuration. The neighbourhoods we considered are presented in Table 5.

The limits of these neighbour bands were chosen somewhat arbitrarily as they would cover an area large enough (up to 90 px) relative to the image sizes. The width of the neighbourhoods were chosen as further bands would be wider and nearer bands would be thinner. For all experiments we chose to sample 5 neighbours from the remote band (i.e., *k* = 5), since we could not find a significant difference in performance when varying *k* (see Section 4.3.1 for details of this experiment).

Growcut parameters. When running the experiments, we limited the number of iterations for one image to 2000. If this number of iterations was reached without convergence, the segmentation was returned in its non-converged form.

Seeds. GrowCut requires a set of initial values (i.e., seeds) to be set by the user. In order to avoid subjectivity in setting these seeds, we created the seeds algorithmically. In this algorithm one vertical seed (i.e., a line of pixels running down the same column of the image) is generated for the background and one for the foreground. These seeds are chosen in a way that, out of all possible vertical seeds for that region (background/foreground), they have the maximum height. The seeds are then scaled to a percentage of their height (we used 75% in our experiments).

Handling 3D images. 3D volumes can be interpreted in two different ways: on one hand, they can be seen as a stack of 2D images slices, and on the other hand, they can be seen as full 3D volumes, in which case we require that all dimensions have the same scale (isovoxels). These two ways of interpreting the 3D volumes map to different ways of extending the GrowCut algorithm to 3D volumes. For a stack of slices interpretation, it is natural to segment each 2D slice independent of the others and reassemble the slices into a 3D volume. For a full 3D interpretation, we can leverage the 3D formulations of CA neighbourhoods. On our experiments, we have used both formulations and used one seed for each slice:

- Stitch: we segment each slice individually and stack the resulting segmentations.
- Full: we use the neighbourhoods’ definition for 3 dimension, i.e., a cell in the CA corresponds to a voxel in the 3D volume.

Performance measures. The performance measures used in this experiment are divided into two categories: supervised and unsupervised measures. Some of the supervised performance measures are: Precision, Recall, False Positive error (FP), False Negative error (FN), True Positive error (TP), Jaccard index, Dice overlap measure, Probabilistic Rand Index (PRI), Variation of Information (VoI), Global Consistency Error (GCE) and Boundary Displacement Error (BDE) [40,41].

The Dice score reports segment overlap as the ratio between the total overlap area and the sum of the two segments’ individual areas. When the two segments are the predicted and ground truth segment, Dice penalizes both false positive and false negative segmentations. Precision and Recall offer a more clear view of the segmentation error, since they only penalize false positive or false negative segmentations, respectively. The Dice score can be derived from precision and recall scores as their harmonic mean [42].

Discrepancy, foreground error, background error, and uniformity are unsupervised measures used to measure the performance of the proposed method. These measures summarize properties of the segmented regions, without taking into account the ground truth segmentation. Discrepancy measures the grey-level difference between the original image and the output image after thresholding. It was proposed to evaluate thresholding-based segmentation techniques that separate the foreground object from the background [43]. The foreground error and the background error quantify the intensity homogeneity of the foreground and background regions, respectively. More specifically, this is calculated as the sum of squared differences between the intensities of the pixels in a region and the mean intensity in the same region. The uniformity measure is a convex combination of foreground and background error, with coefficients given by the proportion of foreground and background pixels, respectively.

After using these, we considered other performance measures too, as they have different relevance in qualitative analysis of segmentation. Some of these metrics are based on the overlap between the resulted segmentation and the ground truth (e.g., TPR, Adjusted Rand Index, Global Consistency Error, Mutual Information, Cohen’s Kappa). Although overlap is an important aspect of segmentation quality, overlap-based metrics do not emphasize the fidelity of the segmentation’s contour. Therefore, the distance-based metrics (e.g., Mahalanobis Distance, (Average) Hausdorff Distance) can be considered, since they are able to highlight the differences between two segmentation’s profiles.

In addition, the performance measures borrowed from supervised classification domain (e.g., TP, TN, etc.), work well in the case of balanced classification problems, where each class has the same number of examples. In the case of segmentation, it is common to have a disproportionate number of training data in one class (background pixels) compared to the other class. Therefore, in addition to the classic accuracy metrics, other performance measures are taken into account (Area Under ROC Curve, Adjusted Rand Index, Mutual Information, Recall, Variation of Information, Over/Under Segmentation Entropy [44]).

### 4.3. Results

#### 4.3.1. Experiment 1

The first experiment aimed to validate the proposed Band-Based GrowCut by comparing against ground truth (proving the confidence of good segmentation ability) for the following image sets: 2D (synthetic, Berkeley, 2D MRI) and 3D (3D MRI).

As a first step of our analysis, two aspects have been considered: the influence of the number of neighbours sampled from a remote band (the *k* parameter) and the band’s width over the segmentation process.

In order to pick a value for the *k* parameter, we ran an experiment where we evaluated the performance of different *k* values across all four different remote bands and three datasets (we considered only 2D images: synthetic, Berkeley, 2D MRI). Table 6 shows the average DICE values for *k* values of 5 neighbours, 20 neighbours, and 1.5% of the number of neighbours in the remote band.

In the latter scenario (1.5% of neighbours), the *k* parameter changes with the size of the employed neighbour band. The results suggest that there is no significant difference in performance between the three *k* values. For this reason and for a reduced computational effort, we chose to fix *k* = 5.

**Figure 4:**
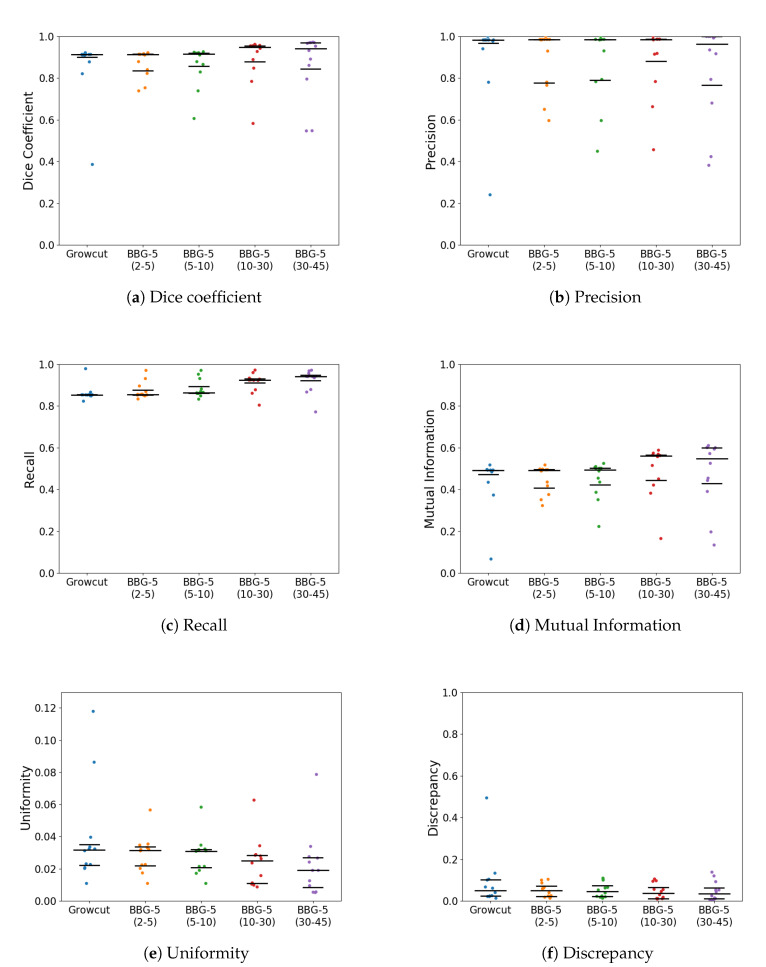
Performance of the evaluated algorithms on the Synthetic images. Median, 1st quartile and 3rd quartile values are shown in black.

**Figure 5:**
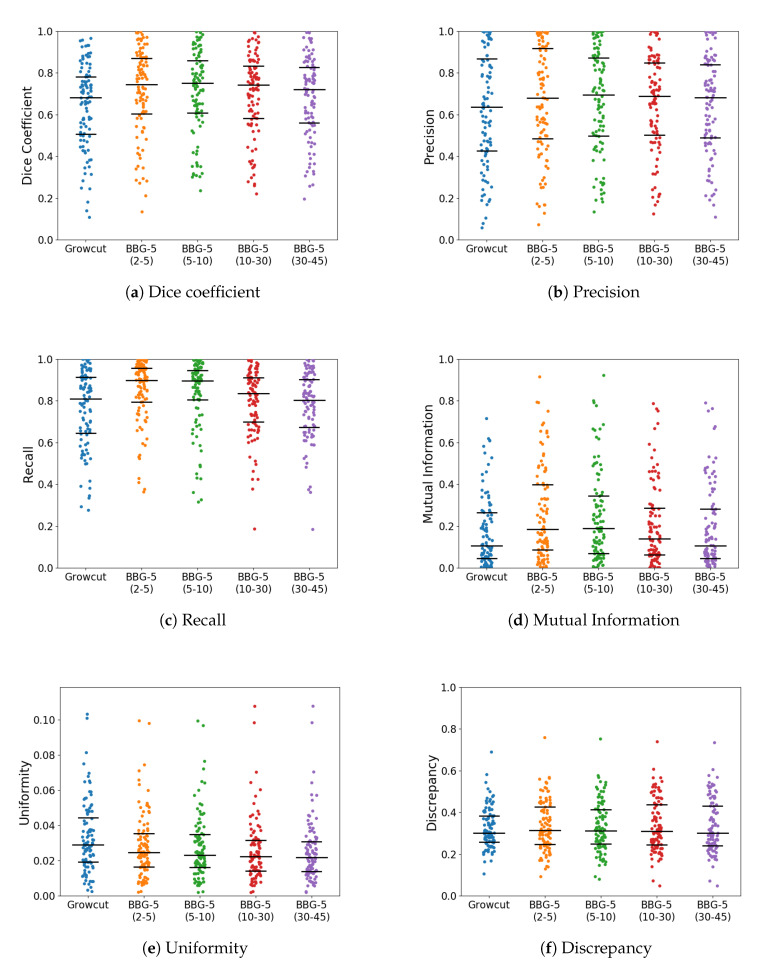
Performance of the evaluated algorithms on the Berkeley images. Median, 1st quartile and 3rd quartile values are shown in black.

**Figure 6:**
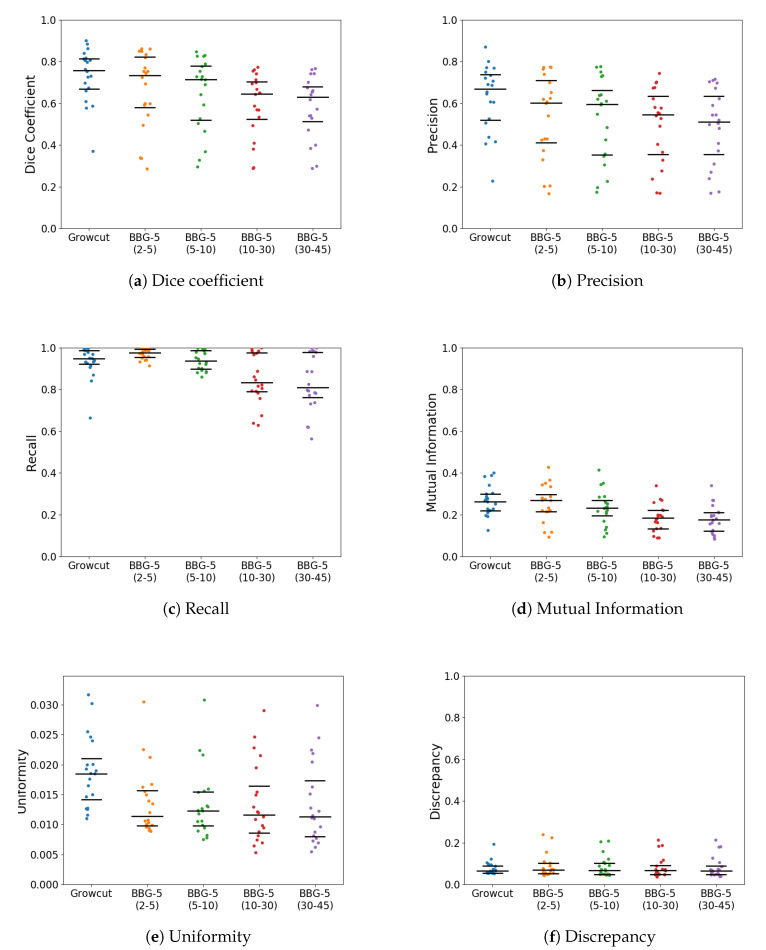
Performance of the evaluated algorithms on the MMWHS 2D MRI images. Median, 1st quartile and 3rd quartile values are shown in black.

**Figure 7:**
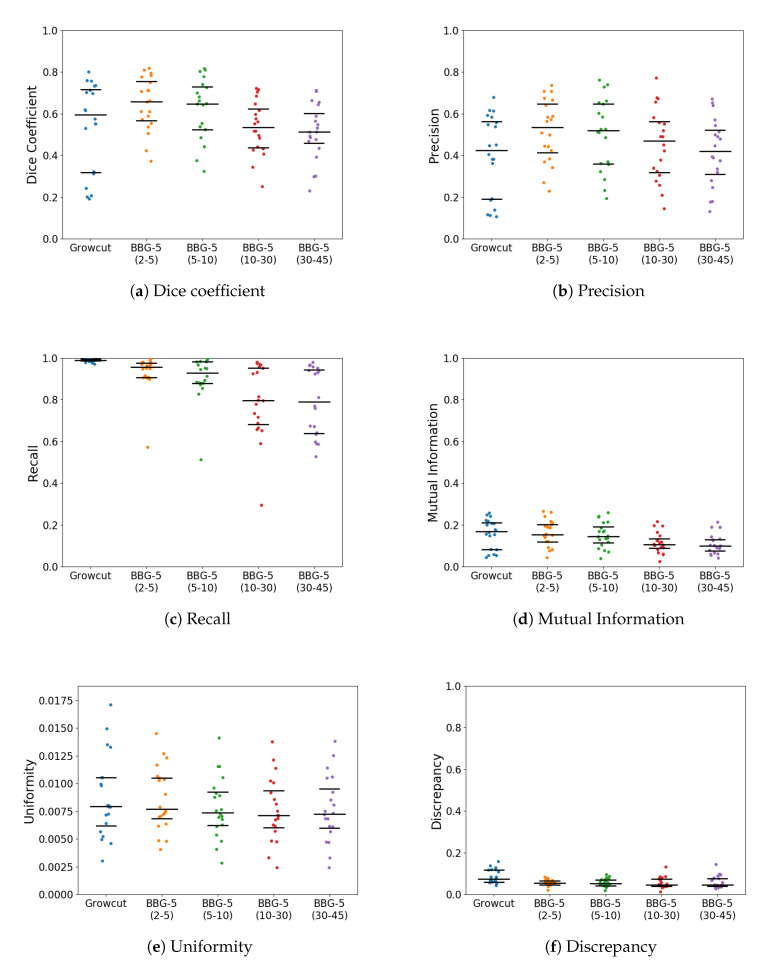
Performance of the evaluated algorithms on the MMWHS 3D MRI images (using a Stitch neighbourhood, i.e., each slice was segmented individually). Median, 1st quartile and 3rd quartile values are shown in black.

**Figure 8:**
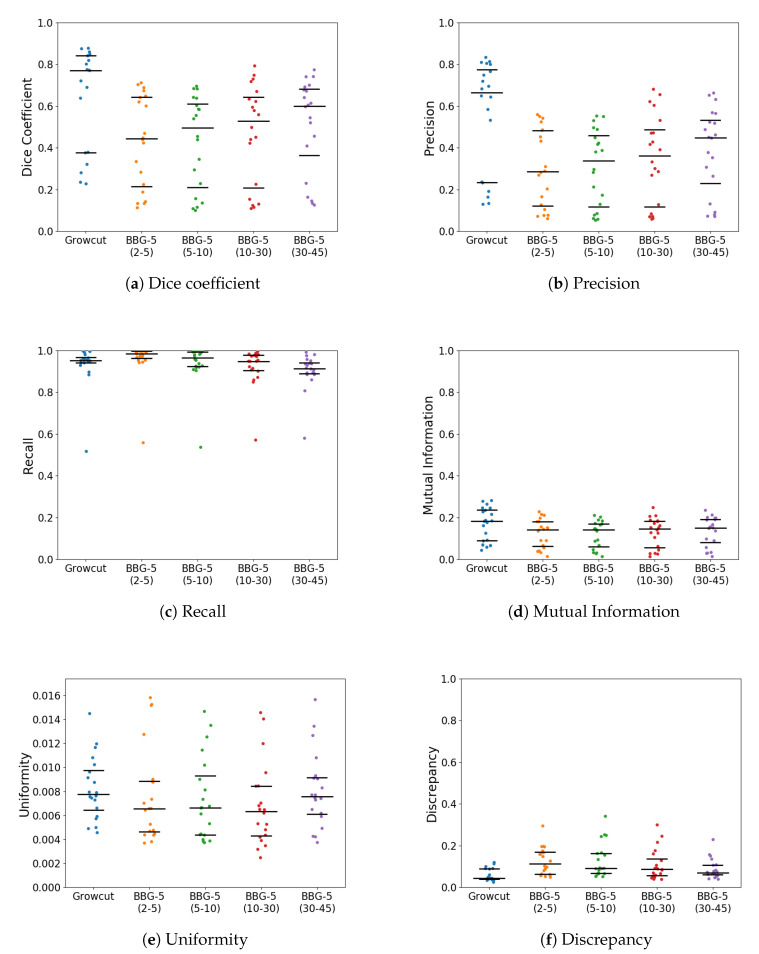
Performance of the evaluated algorithms on the MMWHS 3D MRI images (using a Full neighbourhood, i.e., full 3D extensions of the neighbourhoods systems). Median, 1st quartile and 3rd quartile values are shown in black.

**Figure 9:**
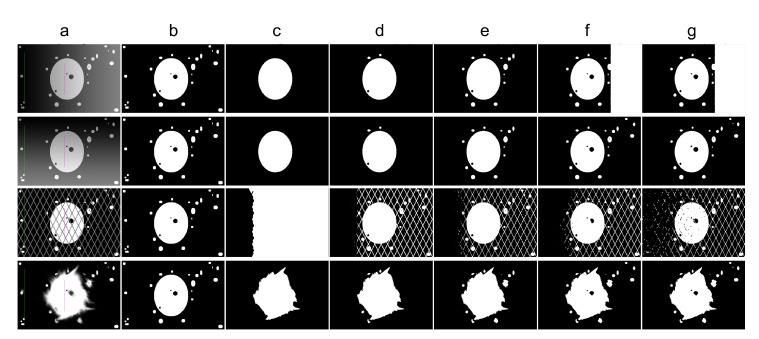
Sample images from the synthetic dataset along with the segmentation results for different algorithms. Legend: (**a**) input image along with the foreground seed (magenta) and background seed (green); (**b**) ground truth; (**c**) Classical GrowCut; (**d**) BBG-5 (2, 5); (**e**) BBG-5 (5, 10); (**f**) BBG-5 (10, 30); (**g**) BBG-5 (30, 45).

**Figure 10:**
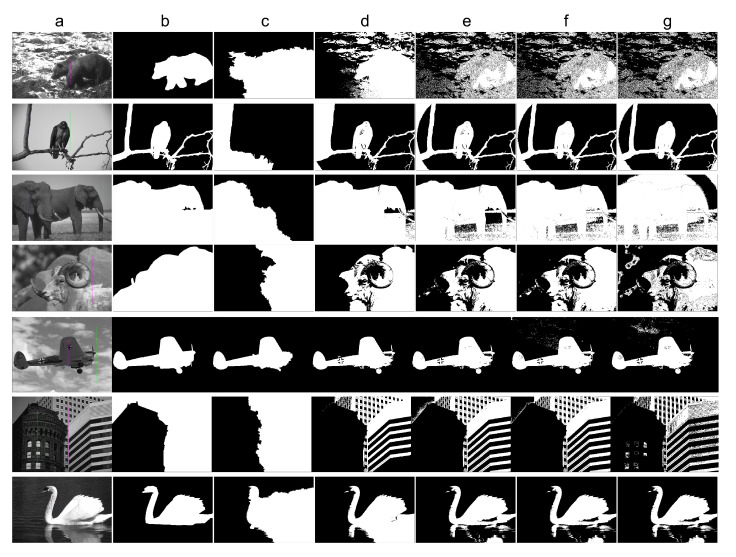
Sample images from the Berkeley dataset along with the segmentation results for different algorithms. Legend: (**a**) input image along with the foreground seed (magenta) and background seed (green); (**b**) ground truth; (**c**) Classical GrowCut result; (**d**) BBG-5 (2, 5) (**e**) BBG-5 (5, 10); (**f**) BBG-5 (10, 30); (**g**) BBG-5 (30, 45).

**Figure 11:**
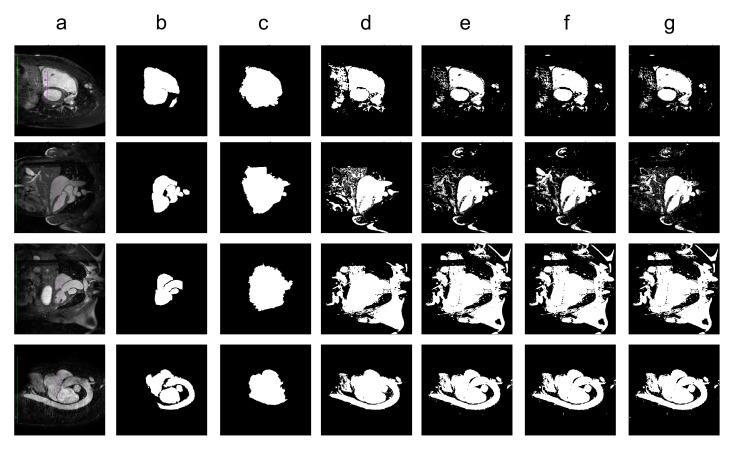
Sample images from the 2D MRI dataset along with the segmentation results for different algorithms. Legend: (**a**) input image along with the foreground seed (magenta) and background seed (green); (**b**) ground truth; (**c**) Classical GrowCut result; (**d**) BBG-5 (2, 5) (**e**) BBG-5 (5, 10); (**f**) BBG-5 (10, 30); (**g**) BBG-5 (30, 45).

With *k* parameter fixed, we were interested in validating the performance of Band-Based GrowCut in segmenting various images (2D and 3D, synthetic or real). Next figures show the DICE, Precision, Recall, Mutual Information, Uniformity and Discrepancy results obtained on the Synthetic (Figure 4), Berkeley (Figure 5), 2D MRI (Figure 6) and 3D MRI datasets (Figure 7 and Figure 8). Figure 9, Figure 10 and Figure 11 show sample images from 2D datasets along with the segmentations obtained using the different algorithms.

These results suggest a generally good segmentation performance of GrowCut when using a hybrid, band-based neighbourhood (BBG). The segmentation quality obtained by various neighbourhoods (classic or band-based) are somehow similar, validating that the proposed approach can solve the considered problem. If we take into account the segmentation quality, two bands are better (5–10 and 30–45) for the majority of experiments, while if we consider the algorithm complexity 5–10 band induces the best results. The obtained segmentations emphasize a special ability of the proposed extended neighbourhood to grow regions that bring together local and global information. In addition to the main region of interest, the proposed algorithm can identify small or particular segments of the image to be segmented (e.g., in Figure 9 BBG identifies the small foreground elements, while in Figure 10 the mark on the plane was also segmented). Furthermore, in the case of MMWHS 2D MRI dataset (see Figure 11), we can see qualitatively that the various hybrid neighbourhood approaches tend to better delimit contours of the heart structures of interest.

#### 4.3.2. Experiment 2

The second experiment is aimed to compare BBG with classical GrowCut in terms of segmentation quality and temporal complexity (running time or number of iterations) for considered image datasets: 2D (synthetic, Berkeley, 2D MRI) and 3D (3D MRI).

In some scenarios, we can observe an improvement in terms of segmentation quality generated by the extended neighbourhood. The remote cells included in the proposed neighbourhood increase the computational complexity of GrowCut in terms of number of operations. To this end, we have analysed several metrics related to the computational effort: the number of iterations performed by GrowCut until it converges (Iteration count) and the required time for performing these iterations (Segmentation time). The obtained values are presented in Table 7, Table 8, Table 9 and Table 10.

We can observe that segmentation time for different neighbourhood varies with dataset. For example, in the 2D MRI dataset, information is evenly spread in close neighbourhood to pixels. In other words, for small neighbourhoods such as $N^{\left(2,5\right)}$, the nature of label propagations is, on average, the same as for the standard neighbourhoods. This is due to the nature of the images, which contain large areas of heart structures, with even intensity. Thus, the CA is much more prone to propagate labels from multiple neighbours (both remote and local). This increases the total segmentation time for small neighbourhoods. Conversely, in these situations, the CA converges in less iterations due to the uniformity of the information in small neighbourhoods around pixels.

Similarly, for further neighbourhoods (such as $N^{\left(5,10\right)}$, $N^{\left(10,30\right)})$, information available in these neighbours is not as uniform, determining a larger number of iterations to convergence, but with faster overall segmentation times (please refer to Table 9 for value). In the $N^{\left(30,45\right)}$ case, we can see a fast segmentation time along with small number of iterations to convergence, which is explained by the sparsity of label propagations from these neighbours. Similar computational performance patterns can be observed for the Synthetic and Berkeley datasets (Table 7 and Table 8).

In the 2D case, most runs converge within 2000 iterations. This is not the case, though, for the 3D dataset. We observe that the increase in dimension determines much larger segmentation times (please refer to Table 10 for value). We attribute this to both the increase in available information along all dimensions, and to the large increase in neighbour count (both local and remote).

In order to directly compare the performance of BBG configurations with GrowCut’s performance, we also analyzed the image-level difference in performance for these algorithms (i.e., increase/decrease in performance of each image when processed using a BBG configuration relative to GrowCut). These results are consistent with our other results, yet they show a smaller variance in the measured performance metrics, since they factor out the influence of individual images. This set of results is available in the Supplementary Material (Supplementary Figures S1–S5).

#### 4.3.3. Experiment 3

In this experiment we investigated the presence of a butterfly effect by assessing whether by changing the initial conditions of the system (i.e., the neighbourhood) we can determine a change in image segmentation properties. In other words, we were interested in whether the small change in neighbourhood system can propagate to the high-level output semantics. To this end, we performed two sets of measurements, one at the image segmentation properties level, where we analyze the characteristics of the output, and one at the cellular automata level, where we assess whether the change in neighbourhood system determines a change in low-level automata behavior. The latter measurement was performed to determine whether the observed macro behavior is concurrent with a change in low-level behavior, that can be more easily linked to the change in initial conditions. We performed these analyses on synthetic images, since these show the least annotation ambiguity with respect to the segmentation property measured.

At image segmentation level, we investigated the segmentation granularity as the representative property. Specifically, we measured over- and under-segmentation as quantified by Over Segmentation Entropy (OSE) and Under Segmentation Entropy (USE) [44] averaged across the dataset. OSE is defined as conditional entropy of the segmentation given the ground truth:

(6) $$OSE=-\sum_{S,T}P\left(T,S\right)logP\left(S|T\right)$$

where *S* is the segmentation and *T* is the respective ground truth. Similarly, USE is defined as the conditional entropy of the ground truth given the segmentation [44]:

(7) $$USE=-\sum_{S,T}P\left(T,S\right)logP\left(T|S\right)$$

Our results on synthetic images are presented in Table 11. We chose this dataset, since we had a properly labeled ground truth only for these in order to measure over- and under-segmentation. On the macro level, the results of our experiments indicate that BBG shows a dominant oversegmentation behavior—the ratio between the OSE and the USE varies between 202% and 348% for BBG, while the same ratio is 22% for the Classical GrowCut. Moreover, we observe that the under segmentation behavior is less pronounced as the distance to the band-based neighbourhood increases, reaching USE values that are below those obtained by Classical GrowCut.

In order to inspect the micro-level behavior, we ran an experiment where we registered the total number of propagated levels (wins) across a run of the algorithm and the nature of this label propagations. Specifically, we investigated whether the label propagation had at its source a neighbour in the standard neighbourhood, or one in a remote band. Moreover, we also registered the number of label propagations that were *overridden* by a neighbour in the far band, i.e., a local neighbour would have propagated the label in absence of remote neighbours.

These metrics quantify the behavior of the algorithm in terms of its most basic operation: the label propagation. We can thus extract information about the preference towards certain neighbours in the label propagation decision process.

Our results are presented in Table 12 and show that the number of total wins decreases with the distance of the remote neighbours to their respective cell. Another insight is that, when employed, the remote neighbours are responsible for 48% of the total wins (on average); these numbers do not seem to depend on the employed remote band. The expected number of remote neighbour wins in a uniformly random scenario would be close to 38%, equal to the fraction of remote neighbours to local ones. Our results show values above this reference, suggesting the existence of a preference towards propagating labels through remote neighbours.

These results show that, by inducing a small change in the initial conditions of the system (i.e., the neighbourhood), we achieve a change in high-level output semantics, implying the existence of a butterfly effect. Moreover, this effect on output segmentation granularity can also be observed at the level of label propagations, the most fundamental operation of the system.

These results suggest that, by having small changes in the initial conditions of the performed task, we can induce major changes in the final outcome of the segmentation. This behaviour translates into the appearance of a butterfly effect, due to the enhancement of a cellular automaton with chaotic features, that brings it closer to the edge of chaos.

## 5. Conclusions

In this work we introduced Band-Based GrowCut, an extension to GrowCut—an interactive, image segmentation algorithm based on cellular automata. The topology and neighbourhood represent important initial conditions of a CA. In the context of image segmentation, we show how small changes in these initial conditions have a significant impact on the final outcome. These changes are realized by adding new distanced cells to the classical neighbourhood in order to collect extra information, which afterwards propagates itself through the entire system. The effect of this propagation can be seen in the regions that the enhanced cellular automata can detect, in addition to the main object of interest. We leveraged developments in cellular automata to endow the algorithm with global information at each step in the segmentation process.

Furthermore, we analyzed the proposed GrowCut extension at different levels. We measured the oversegmentation effect the inclusion of remote neighbours has on the segmentation, as well as the effect on the CA’s dynamics. We evaluated the algorithm on a series of 2D and 3D heterogeneous segmentation tasks and compared it against classical GrowCut, showing that our proposed extension outperforms it on synthetic and natural images. Finally, our results suggest that, by having small changes in the initial conditions of the performed task, we can induce major changes in the final outcome of the segmentation.

The current work is part of a more complex one, dedicated to the analysis of how each component of a CA affects its performance in solving a particular task. While the proposed approach is focusing on analysing the role of the neighbourhood’s topology, as future work we plan to investigate the other aspects that could influence the performance of our Band-Based GrowCut algorithm: the label propagation principles and the CA’s initial state governed by the seeds in the case of our Band-Based GrowCut algorithm.

## Figures and Tables

**Table 1:** Initial states of the GrowCut CA cells in our example. BG—background, FG—- foreground.

	A	B	C	D
Intensity	1.0	0.1	1.0	0.1
Strength	1.0	0.0	0.0	1.0
Label	BG	-	-	FG

**Table 2:** Strengths of all cell attacks during the first iteration. Columns represent attacking cells and rows represent defending cells. Bold values represent successful attacks.

Defending Cell	A	B	C	D	Defense Strength
A		0.0	-	-	1.0
B	0.1		0.0	-	0.0
C	-	0.0		0.1	0.0
D	-	-	0.0		1.0

**Table 3:** States of the GrowCut CA cells after the first iteration. BG—background, FG—foreground.

	A	B	C	D
Intensity	1.0	0.1	1.0	0.1
Strength	1.0	0.1	0.1	1.0
Label	BG	BG	FG	FG

**Table 4:** Strengths of all cell attacks during the second iteration. No attacks were successful, therefore the algorithm converged.

Defending Cell	A	B	C	D	Defense Strength
A		0.01	-	-	1.0
B	0.1		0.01	-	0.1
C	-	0.01		0.1	0.1
D	-	-	0.01		1.0

**Table 5:** GrowCut configurations considered for our experiments. The columns describe the type of local and remote neighbourhoods used, as well as the number of neighbours sampled from the remote band. The remote bands are coded by specifying their neighbourhood type (e.g., Moore) and, in parantheses, the radii they span. For example, Moore (2,5) refers to a neighbour band spanning radii 2 through 5.

	Standard Neighbourhood	Remote Neighbourhood	Neighbours Sampled from Remote Neighbourhood
Classical GrowCut	Moore	-	-
BBG-5 (2, 5)	Moore	Moore (2, 5)	5
BBG-5 (5, 10)	Moore	Moore (5, 10)	5
BBG-5 (10, 30)	Moore	Moore (10, 30)	5
BBG-5 (30, 45)	Moore	Moore (30, 45)	5

**Table 6:** Average DICE coefficient for different *k* values, across different datasets.

*k*	Synthetic	Berkeley	2D MRI
5	0.870	0.687	0.595
20	0.858	0.684	0.589
1.5% of remote neighbour band	0.854	0.683	0.592

**Table 7:** Results for Synthetic dataset (average computational complexity metrics).

Method	Iteration Count	Segmentation Time (Seconds)
GrowCut	493.67	6.00
BBG-5 (2, 5)	249.42	7.11
BBG-5 (5, 10)	302.75	5.65
BBG-5 (10, 30)	371.33	5.96
BBG-5 (30, 45)	303.00	6.27

**Table 8:** Results for Berkeley dataset (average computational complexity metrics).

Method	Iteration Count	Segmentation Time (Seconds)
GrowCut	515.68	6.12
BBG-5 (2, 5)	611.34	8.64
BBG-5 (5, 10)	765.47	7.04
BBG-5 (10, 30)	731.98	7.27
BBG-5 (30, 45)	614.77	7.35

**Table 9:** Results for 2D MRI dataset (average computational complexity metrics).

Method	Iteration Count	Segmentation Time (Seconds)
GrowCut	684.70	6.69
BBG-5 (2, 5)	388.15	7.74
BBG-5 (5, 10)	485.40	6.18
BBG-5 (10, 30)	471.45	6.15
BBG-5 (30, 45)	381.45	6.31

**Table 10:** Results for 3D MRI dataset (average computational complexity metrics). Stitch: each slice of the image was segmented individually as a 2D image; Full: a 3D neighborhood was used (further detailed in Section 4.2).

Method	Iteration Count	Segmentation Time (Seconds)
Stitch GrowCut	1031.60	265.42
Stitch BBG-5 (2, 5)	1568.85	285.29
Stitch BBG-5 (5, 10)	1996.95	350.20
Stitch BBG-5 (10, 30)	2000.00	345.02
Stitch BBG-5 (30, 45)	1987.65	365.08
Full GrowCut	1031.60	267.07
Full BBG-5 (2, 5)	2000.00	520.46
Full BBG-5 (5, 10)	2000.00	473.96
Full BBG-5 (10, 30)	1850.05	484.71
Full BBG-5 (30, 45)	1151.55	4399.69

**Table 11:** Over and Under Segmentation Entropy measured on our synthetic dataset.

Algorithm	OSE	USE	OSE/USE Ratio
Classical GrowCut	0.038	0.175	22%
BBG-5 (2, 5)	0.389	0.205	190%
BBG-5 (5, 10)	0.420	0.208	202%
BBG-5 (10, 30)	0.445	0.146	305%
BBG-5 (30, 45)	0.466	0.134	348%

**Table 12:** Number of label propagations classified by their source (local, remote, or overridden by remote)—Synthetic dataset.

Method	GrowCut	BBG-5 (2, 5)	BBG-5 (5, 10)	BBG-5 (10, 30)	BBG-5 (30, 45)
Local Wins	100%	42%	47%	63%	58%
Remote Wins	0%	30%	26%	18%	18%
Remote Overrides	0%	28%	27%	19%	24%
Total Wins ($10^{6}$)	44.76	15.93	33.31	26.10	16.69

## Supplementary Materials

The following are available at https://www.mdpi.com/1099-4300/22/9/1028/s1.
